# ID 205 - Criação de um Aplicativo de Manejo Clínico para Indivíduos com Suspeita de Dengue

**DOI:** 10.5327/2237-9622.2025.v34s1.205

**Published:** 2025-11-25

**Authors:** Marcos Oliveira Silva, Gabriela Oliveira

**Keywords:** tecnologia, acesso à informação, saúde, dengue

## Abstract

**Introdução:**

A aplicação de tecnologias da informação na área da saúde cresce de maneira exponencial, permitindo avanços significativos na medicina, como o tratamento de pacientes e o registro eletrônico de suas histórias clínicas. Tais tecnologias estão transformando a forma como os profissionais de saúde oferecem atendimento, alcançando localidades remotas, aprimorando suas habilidades e democratizando o acesso à informação em saúde (MLA, 2023). Segundo o Instituto Brasileiro de Geografia e Estatística (IBGE), 83% da população brasileira estava conectada à internet em 2023, sendo o smartphone o principal meio de acesso à internet, com 97% de preferência entre os usuários. O desenvolvimento de aplicativos móveis na área da saúde pode se revelar extremamente valioso para os profissionais da saúde, especialmente para aqueles que trabalham em regiões com acesso limitado à informação técnica e oportuna. O presente estudo teve como objetivo geral a elaboração de um aplicativo para smartphones, para ser utilizado por profissionais da saúde em indivíduos com suspeita de dengue, e como objetivos específicos a seleção de tecnologias de código aberto e a conversão dos protocolos clínicos disponíveis em linguagem acessível.

**Método:**

Trata-se de uma pesquisa aplicada em que se desenvolveu um aplicativo móvel para profissionais de saúde. O aplicativo fornecerá informações oportunas e protocolos clínicos para os profissionais da saúde no atendimento a pacientes com suspeita de dengue, baseando-se em documentos elaborados pelo Ministério da Saúde – uma vez instalado, poderá ser utilizado off-line. O conteúdo do aplicativo foi extraído das documentações produzidas pelas equipes técnicas do Ministério da Saúde e disponibilizadas por meio do Portal da Saúde do governo federal. A metodologia para o desenvolvimento do aplicativo envolveu a atualização das tecnologias de programação, utilizando ferramentas de código aberto como PHONEGAP, HTML5, CSS3 e JQUERY.

**Resultados:**

Após a produção do aplicativo, ele foi testado e validado pela área técnica estadual para utilização no manejo clínico de indivíduos com sintomas de dengue. Foi possível verificar maior disseminação e democratização da informação em saúde para a população e profissionais da área; possibilitou o funcionamento como uma ferramenta adicional de apoio aos profissionais de saúde no cuidado e na prevenção de doenças, especialmente em regiões com ou sem conectividade; e, por utilizar tecnologias de programação de código aberto, o aplicativo não gera custos adicionais para o desenvolvedor e pode ser atualizado a qualquer momento pela equipe técnica.

**Conclusão:**

A produção de aplicativos com foco em facilitar e aprimorar o atendimento e o acesso à saúde tem sido cada vez maior e mais democrática (Gondim ., 2024; Assis ., 2024; Mendes ., 2022; Balaji ., 2022). A informação é um recurso valioso para qualquer instituição, e o uso de tecnologias de informação é crucial para profissionais e gestores de saúde que buscam superar barreiras geográficas, reduzir custos e promover a disseminação e a democratização da informação em saúde em benefício dos cidadãos, além de possibilitar a utilização dos dados em pesquisas clínicas, bem como o desenvolvimento de políticas de saúde mais eficazes e de estratégias eficientes no cuidado oportuno ao paciente, baseado em evidências.

